# Associations between neurological examination at term-equivalent age and cerebral hemodynamics and oxygen metabolism in infants born preterm

**DOI:** 10.3389/fnins.2023.1105638

**Published:** 2023-03-02

**Authors:** Gabriel Côté-Corriveau, Marie-Noëlle Simard, Olivia Beaulieu, Rasheda Arman Chowdhury, Marie-Michèle Gagnon, Mélanie Gagnon, Omar Ledjiar, Catherine Bernard, Anne Monique Nuyt, Mathieu Dehaes, Thuy Mai Luu

**Affiliations:** ^1^Research Center, Sainte-Justine University Hospital Center, Montreal, QC, Canada; ^2^Department of Epidemiology, Biostatistics and Occupational Health, Faculty of Medicine, McGill University, Montreal, QC, Canada; ^3^Department of Pediatrics, Sainte-Justine University Hospital Center, University of Montreal, Montreal, QC, Canada; ^4^School of Rehabilitation, University of Montreal, Montreal, QC, Canada; ^5^Institute of Biomedical Engineering, University of Montreal, Montreal, QC, Canada; ^6^Unité de Recherche Clinique Appliquée, Sainte-Justine University Hospital Center, Montreal, QC, Canada; ^7^Department of Radiology, Radio-Oncology and Nuclear Medicine, University of Montreal, Montreal, QC, Canada

**Keywords:** frequency-domain near infrared spectroscopy, diffuse correlation spectroscopy, preterm birth, brain development, neurological examination, cerebral blood flow, cerebral oxygen metabolism

## Abstract

**Background:**

Infants born at 29–36 weeks gestational age (GA) are at risk of experiencing neurodevelopmental challenges. We hypothesize that cerebral hemodynamics and oxygen metabolism measured by bedside optical brain monitoring are potential biomarkers of brain development and are associated with neurological examination at term-equivalent age (TEA).

**Methods:**

Preterm infants (*N* = 133) born 29–36 weeks GA and admitted in the neonatal intensive care unit were enrolled in this prospective cohort study. Combined frequency-domain near infrared spectroscopy (FDNIRS) and diffuse correlation spectroscopy (DCS) were used from birth to TEA to measure cerebral hemoglobin oxygen saturation and an index of microvascular cerebral blood flow (CBF_*i*_) along with peripheral arterial oxygen saturation (SpO_2_). In combination with hemoglobin concentration in the blood, these parameters were used to derive cerebral oxygen extraction fraction (OEF) and an index of cerebral oxygen metabolism (CMRO_2*i*_). The Amiel-Tison and Gosselin Neurological Assessment was performed at TEA. Linear regression models were used to assess the associations between changes in FDNIRS-DCS parameters from birth to TEA and GA at birth. Logistic regression models were used to assess the associations between changes in FDNIRS-DCS parameters from birth to TEA and neurological examination at TEA.

**Results:**

Steeper increases in CBF_*i*_ (*p* < 0.0001) and CMRO_2*i*_ (*p* = 0.0003) were associated with higher GA at birth. Changes in OEF, CBF_*i*_, and CMRO_2*i*_ from birth to TEA were not associated with neurological examination at TEA.

**Conclusion:**

In this population, cerebral FDNIRS-DCS parameters were not associated with neurological examination at TEA. Larger increases in CBF_*i*_ and CMRO_2*i*_ from birth to TEA were associated with higher GA. Non-invasive bedside FDNIRS-DCS monitoring provides cerebral hemodynamic and metabolic parameters that may complement neurological examination to assess brain development in preterm infants.

## Introduction

Infants born between 29 and 36 weeks of gestational age (GA) represent the majority of preterm births (Goldenberg et al., 2008), yet determining the ones at greatest risk of neurodevelopmental impairments remains a challenge (Vohr, 2013; Volpe, 2022). By 2 years of corrected age (CA), 25–45% of these infants have developmental delay (Simard et al., 2012; Cheong et al., 2017). Population-based data indicate that infants born 29–36 weeks GA have 1.4–2.3 times the risk of facing major disabilities affecting their long-term functioning (Moster et al., 2008). A better insight into their brain development is needed to improve early screening strategies and identify those the most vulnerable for neurodevelopmental impairment. Studies using magnetic resonance imaging (MRI) have shown smaller brain volumes and altered white matter microstructure in moderate to late preterm infants at term-equivalent age (TEA) (Kelly et al., 2016; Thompson et al., 2019; Volpe, 2022). While MRI provides high-resolution images of the brain microstructure, it is less feasible for early stratification due to high scanning cost and the high number of moderate to late preterm infants.

An alternative modality to assess brain health at the bedside is cerebral near infrared spectroscopy (NIRS). NIRS has the potential to identify infants at higher risk of neurodevelopmental impairment as early as in the neonatal period (Tataranno et al., 2021). Combining frequency-domain NIRS (FDNIRS) and diffuse correlation spectroscopy (DCS) allows to measure cerebral hemoglobin oxygen saturation (Fantini et al., 1995) and an index of microvascular cerebral blood flow (CBF_*i*_) (Boas and Yodh, 1997), respectively. Using hemoglobin concentration measurements and peripheral arterial oxygen saturation, FDNIRS-DCS parameters can be used to derive cerebral oxygen extraction fraction (OEF) and an index of the cerebral metabolic rate of oxygen consumption (CMRO_2*i*_). This technique was previously used to monitor early brain development in preterm infants (Roche-Labarbe et al., 2010; Lin et al., 2013a,b, 2016), and showed good agreement with gold-standard imaging metrics including MRI and contrast-based optical techniques (Carp et al., 2010; Durduran et al., 2010; Kim et al., 2010; Diop et al., 2011; Buckley et al., 2012; Jain et al., 2014; Milej et al., 2020). Previous studies also examined how NIRS parameters were related with electroencephalographic (EEG) activity in the preterm population, and demonstrated associations between OEF stability and more mature EEG activity (El-Dib et al., 2016), as well as relationships between increase in relative CBF and CMRO_2_ and EEG patterns such as spontaneous physiological neuronal bursts of activity (Nourhashemi et al., 2020).

How FDNIRS-DCS parameters relate to clinical findings in the preterm population is, however, less documented. Data suggest that brain lesions resulting in poorer developmental outcomes in the preterm population are associated with altered cerebral blood flow and oxygen metabolism (Lin et al., 2016; McLachlan et al., 2017). It was shown that even mild intraventricular hemorrhage is associated with lower CBF_*i*_ and CMRO_2*i*_ (Lin et al., 2016), and that severe cases leading to post-hemorrhagic ventricular dilatation have lower CBF before vs after a therapeutic intervention (McLachlan et al., 2017). FDNIRS-DCS also has the potential to inform on brain growth and maturation, including myelination and synaptogenesis (Roche-Labarbe et al., 2012; Lin et al., 2013b,^2016^). As the preterm infant grows, cerebral blood flow and oxygen metabolism would likely increase to reflect brain development and increased brain volumes (Roche-Labarbe et al., 2010, 2012). In a previous study, the increase in brain volumes in preterm infants born before 32 weeks GA was lower than in healthy control fetuses during the third trimester, suggesting impaired brain growth (Bouyssi-Kobar et al., 2016). In the current study, we hypothesized that increases in CBF_*i*_ and CMRO_2*i*_ from birth to TEA would be associated with normal neurological examination at TEA. The aim of the present study was to assess changes in FDNIRS-DCS parameters from birth to TEA and their associations with GA at birth and neurological examination at TEA in infants born between 29 and 36 weeks GA.

## Materials and methods

### Study population

This is a single-center prospective observational cohort study at Sainte-Justine University Hospital Center, a tertiary referral hospital in Montreal, Canada. From August 2018 to April 2020, we consecutively recruited infants born between 29 and 36 6/7 weeks GA who were admitted for at least 48 h at the neonatal intensive care unit. Exclusion criteria were major chromosomal anomaly and congenital malformations, moderate to severe hypoxic ischemic encephalopathy, and neonatal stroke, as these infants already benefit from neurodevelopmental follow-up in specialized clinics; moribund infants; and infants under child protection services due to consent issues. The study (#2019-1936) was approved by the institutional review board of the *Comité d’éthique de la recherche* at Sainte-Justine University Hospital Center, named by the Quebec Government (#FWA00021692) and acts in accordance with Quebec and Canada laws, and the Code of Federal Regulations in the USA. Parental written informed consent was obtained for each patient.

### Data collection

Parents completed a questionnaire for demographic data. Perinatal and neonatal characteristics were retrieved from medical charts. Peripheral arterial oxygen saturation (SpO_2_, %) was measured from preductal pulse oximetry at an upper limb. We used hemoglobin concentration in blood (HGB, g/dl) measured for clinical purposes throughout the hospital stay.

### FDNIRS-DCS monitoring and data analysis

FDNIRS-DCS monitoring was performed with a commercial system (MetaOx, ISS Inc., Champaign, IL, USA) (Buckley et al., 2014; Carp et al., 2017). The system was approved by Health Canada for research purpose only. Optical energy exposure satisfied the American National Standards Institute (ANSI) standard for Safe Use of Lasers and related regulations (ANSI Z136 Standards, 2017). The optical sensor was designed and developed to image the neonatal cortex (Dehaes et al., 2011). The sensor was positioned on the middle frontal location of the head and signals were recorded at least 5 times by repositioning the sensor slightly to account for local inhomogeneities. Left and right frontal locations were assessed in sensitivity analyses. The source-detector separations were 10, 15, 20, and 25 mm for FDNIRS, and 22 mm for DCS (eight collocated detecting optical fibers). This geometry was optimized to probe the brain and minimized signal contamination due to head curvature (Dehaes et al., 2011). Analysis was performed offline by averaging middle frontal values. Data quality assessment and data rejection were performed based on published criteria prior to averaging (Roche-Labarbe et al., 2010, 2012; Lin et al., 2013a; Dehaes et al., 2014, 2015). FDNIRS and DCS systems have a distinct data quality assessment. Rejection criteria included data affected by hair, inadequate head-sensor coupling, bending of the probe, and artifacts due to motion (Roche-Labarbe et al., 2010).

Brain monitoring was performed from the first week of age to TEA, defined as between 37 and 40 weeks of post-menstrual age. The number of monitoring sessions varied between neonates and depended on GA and the length of hospital stay. Each session required 15–20 min. For each patient and FDNIRS-DCS parameter, temporal change was calculated by the difference between the value at (or near) TEA and the value measured at the first visit normalized by the temporal period that separated the two measurements (in weeks).

Absolute cerebral oxygen hemoglobin saturation (SO_2_, %) was calculated by the ratio between oxyhemoglobin (HbO_2_, μmol/L) and total hemoglobin (HbT, μmol/L) derived from FDNIRS signals. OEF was defined by the ratio between the arterio-venous O_2_ saturation difference (SpO_2_ – SvO_2_) and SpO_2_, where SvO_2_ was estimated *via* an arterial:venous contribution ratio of 0.25:0.75 as in previous studies (Watzman et al., 2000; Roche-Labarbe et al., 2012; Lin et al., 2013a; Dehaes et al., 2014, 2015). CBF_*i*_ was derived from DCS signals (Boas and Yodh, 1997; Cheung et al., 2001). The calculation of CMRO_2*i*_ was performed *via* the Fick’s principle (Kety and Schmidt, 1948) as previously described (Roche-Labarbe et al., 2012; Lin et al., 2013a,b; Dehaes et al., 2014, 2015) and estimated by the product of CBF_*i*_, HGB, OEF, and the theoretical maximum oxygen carrying capacity (γ = 1.39 ml O_2_/g of HGB). These calculations were performed in MATLAB (Mathworks, Natick, MA, USA).

### Neurological examination

At TEA, the Amiel-Tison and Gosselin Neurological Assessment (ATGNA) was performed by a trained member of the research team blinded to the medical history and FDNIRS-DCS results. The ATGNA is a standardized 5 min exam consisting of 35 items evaluating head growth, cranial sutures, alertness, passive muscle tone of the limbs and axis, and axial motor activity. Results are synthesized in clusters to provide a final categorization: optimal (normal) exam defined as no neurological sign, or non-optimal (abnormal) exam with presence of mild/moderate or severe neurological signs (Gosselin et al., 2005). Previous works have demonstrated high inter-observer reliability (*k* = 0.92) (Simard et al., 2009), stability of results over time (Cochran’s *Q* = 7.224) (Simard et al., 2010), and ability to discriminate preterm born infants with developmental delay (Simard et al., 2011) and suboptimal neuromotor status (Leroux et al., 2013).

### Statistical analysis

Descriptive statistics are reported as means (standard deviations) and proportions. The association of FDNIRS-DCS first measure and temporal changes with GA at birth was assessed using univariate linear regression models. We further examined the effect of sex using multiple linear regression models. We performed logistic regression models to estimate odds ratios (OR) and 95% confidence intervals (CI) for the associations between FDNIRS-DCS parameters and the neurological examination at TEA. Models for the relationship between FDNIRS-DCS parameters at first visit and neurological exam at TEA were adjusted for post-menstrual age at the time of measurement, as it did not systematically occur at the same post-menstrual age for each patient. We performed subgroup analyses stratifying by GA (29–32 vs 33–36 weeks GA) and by sex (females vs males). We also examined how cerebral SO_2_ measures were associated with the neurological examination by performing logistic regression models. In sensitivity analyses, we assessed the association of FDNIRS-DCS temporal changes measured on the left and right frontal regions with GA at birth and with neurological exam at TEA using linear and logistic regression models, respectively. *P*-values of <0.05 were considered statistically significant, and analyses were conducted using SAS version 9.4 (SAS Institute Inc., Cary, NC).

## Results

**FIGURE 1:**
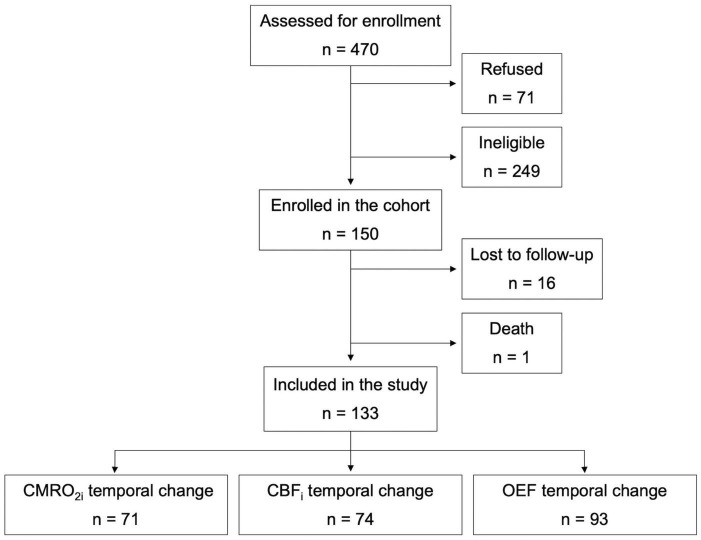
Flow chart of participants.

Among 150 infants initially recruited, 133 completed the neurological assessment at TEA and were included in the study (Figure 1). Participants who were lost to follow-up were comparable to infants who completed the neurological assessment in terms of clinical characteristics, except for higher mean birth weight and birth weight Z-Score (Supplementary Table 1). The cohort was composed of 60 females (45%) and 73 males (55%), with a mean GA of 33 weeks and mean birth weight of 1831 grams. Two neonates included in the study had a suspected mild hypoxic ischemic encephalopathy. At TEA, 71 (53%) infants had an abnormal neurological examination. Infants with normal vs abnormal neurological examination were comparable for mean gestational age, birth weight, head circumference, antenatal corticosteroid, and surfactant administration (Table 1). There were more multiple births among those with an abnormal neurological examination (41% vs 19%), as well as more males (62% vs 47%), and urgent cesarean sections (56% vs 40%).

**TABLE 1:** Neonatal characteristics of the study population according to abnormal or normal neurological examination at term-equivalent age.

	Abnormal (*n* = 71)	Normal (*n* = 62)	All participants (*n* = 133)
Gestational age, mean (SD), weeks	33.5 (1.9)	33.1 (1.8)	33.3 (1.9)
Birth weight, mean (SD), *g*	1815.3 (555.4)	1849.0 (546.5)	1831.0 (549.4)
Birth weight Z-score, mean (SD)	−0.7 (1.1)	−0.4 (1.0)	−0.6 (1.1)
Head circumference, mean (SD), cm	29.6 (2.0)	29.7 (2.3)	29.6 (2.1)
Male, *n* (%)	44 (62.0)	29 (46.8)	73 (54.9)
Multiple birth, *n* (%)	29 (40.8)	12 (19.4)	41 (30.8)
Antenatal corticosteroids, *n* (%)	56 (78.9)	51 (82.3)	107 (80.5)
Urgent cesarean section, *n* (%)	40 (56.3)	25 (40.3)	65 (48.9)
Surfactant administration, *n* (%)	12 (16.9)	12 (19.4)	24 (18.0)

SD, standard deviation.

After data quality assessment of FDNIRS and DCS data separately, CMRO_2*i*_ data at TEA were available in 90 patients, for CBF_*i*_ in 92 patients, and for OEF in 98 patients. When considering FDNIRS-DCS temporal change, CMRO_2*i*_ data were available in 71 patients, for CBF_*i*_ in 74 patients, and for OEF in 93 patients. The average time between birth and consent was 4.13 days (SD = 2.21), and the average time between birth and first FDNIRS-DCS measurement was 4.88 days (SD = 2.94). Mean time interval between first and last FDNIRS-DCS measurements was 4.45 weeks (SD = 2.77).

**FIGURE 2:**
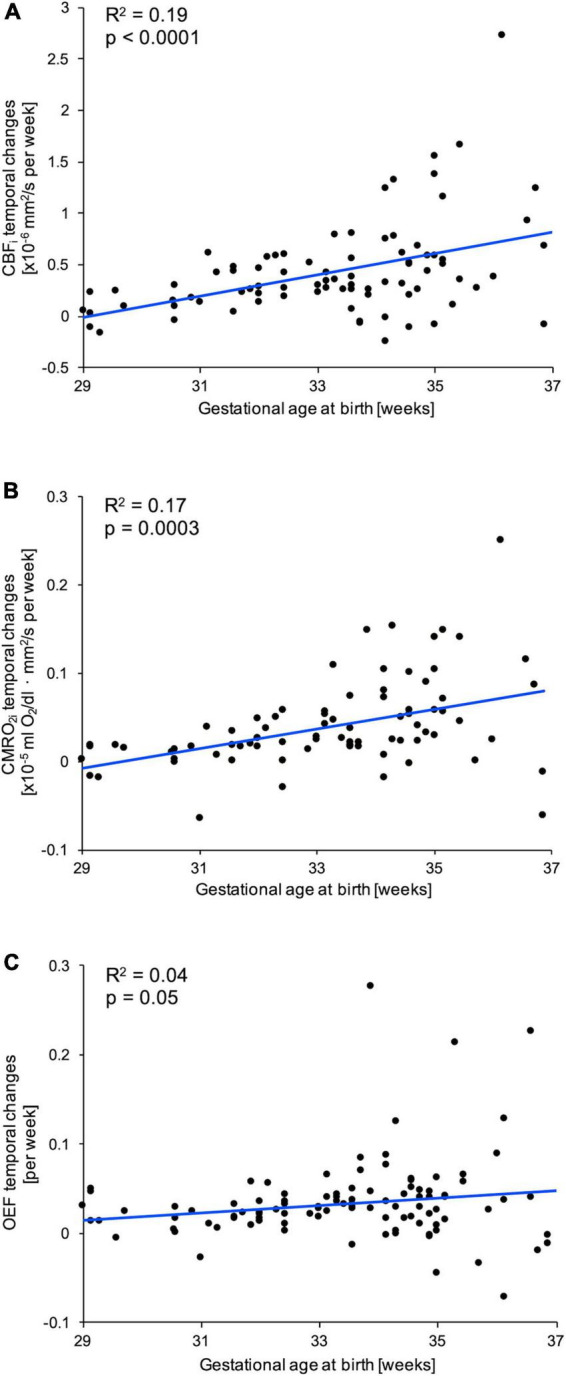
Associations between temporal changes of **(A)** cerebral blood flow index (CBF_*i*_), **(B)** cerebral metabolic rate of oxygen consumption index (CMRO_2*i*_), and **(C)** cerebral oxygen extraction fraction (OEF) with gestational age at birth (GA). For each parameter, a linear fit (blue line) and corresponding correlation coefficient (*R*^2^) and *p*-value are provided.

Steeper increases in CBF_*i*_ (*p* < 0.0001) and CMRO_2*i*_ (*p* = 0.0003) were associated with higher GA at birth (Figure 2). However, there was no significant association between temporal change in OEF and GA (*R*^2^ = 0.04; *p* = 0.05). In contrast, there was no significant association between CMRO_2*i*_ and CBF_*i*_ at first visit and GA at birth. The relationship between OEF first measurement and GA reached statistical significance (*R*^2^ = 0.05, *p* = 0.02), but the magnitude of association was very weak (Supplementary Figure 1). In adjusted linear models, infant sex was not significantly associated with the temporal change in FDNIRS-DCS parameters (CBF_*i*_: *p* = 0.26; CMRO_2*i*_: *p* = 0.38; OEF: *p* = 0.72).

We did not identify any significant association between FDNIRS-DCS parameters and neurological examination at TEA, whether examining OR considering the first and last measurements, or the temporal change in FDNIRS-DCS parameters (Table 2). Stratifying by sex (females vs males) or by GA groups (29–32 vs 33–36 weeks GA) did not change this relationship. There were also no significant associations between cerebral SO_2_ at first and last visits or temporal change in SO_2_ and neurological examination at TEA (Supplementary Table 2).

**TABLE 2:** Associations between FDNIRS-DCS parameters and temporal changes and neurological examination at term-equivalent age.

Odds ratios (95% confidence interval)									
	**First FDNIRS-DCS parameters^a^**			**FDNIRS-DCS parameters at TEA**			**FDNIRS-DCS temporal changes**		
	**OEF**	**CBF_*i*_**	**CMRO_2*i*_**	**OEF**	**CBF_*i*_**	**CMRO_2*i*_**	**OEF**	**CBF_*i*_**	**CMRO_2*i*_**
All participants	0.98 (0.92–1.03)	0.96 (0.90–1.03)	0.97 (0.90–1.03)	0.98 (0.92–1.03)	1.00 (0.96–1.03)	1.01 (0.98–1.05)	1.00 (0.90–1.10)	1.08 (0.96–1.22)	1.07 (0.96–1.20)
**Stratified by gestational age**									
29–32 weeks	0.95 (0.84–1.07)	0.97 (0.86–1.08)	0.87 (0.73–1.03)	0.98 (0.87–1.11)	0.99 (0.93–1.07)	1.03 (0.94–1.12)	2.24 (0.87–5.73)	1.21 (0.75–1.96)	1.79 (0.82–3.91)
33–36 weeks	0.99 (0.92–1.06)	0.96 (0.88–1.05)	0.99 (0.92–1.07)	0.98 (0.92–1.05)	1.00 (0.96–1.04)	1.01 (0.97–1.04)	0.98 (0.88–1.08)	1.06 (0.94–1.20)	1.04 (0.93–1.16)
**Stratified by sex**									
Females	1.02 (0.94–1.10)	1.00 (0.90–1.11)	1.07 (0.94–1.22)	0.97 (0.89–1.05)	0.96 (0.90–1.03)	0.93 (0.86–1.02)	0.88 (0.69–1.11)	1.15 (0.82–1.62)	1.07 (0.82–1.39)
Males	0.94 (0.86–1.03)	0.94 (0.86–1.03)	0.92 (0.84–1.01)	0.99 (0.92–1.07)	1.01 (0.97–1.06)	1.03 (0.99–1.08)	1.07 (0.91–1.26)	1.06 (0.94–1.21)	1.06 (0.94–1.20)

^a^Odds ratios with first FDNIRS-DCS parameters are adjusted for post-menstrual age at time of measurement.

CBF_*i*_, cerebral blood flow indices; CMRO_2*i*_, cerebral metabolic rate of oxygen consumption indices; FDNIRS-DCS, frequency-domain near infrared spectroscopy – diffuse correlation spectroscopy; OEF, oxygen extraction fraction; TEA, term-equivalent age.

In sensitivity analyses, models considering FDNIRS-DCS measurements on the left and right frontal regions yielded results that were comparable to our main analyses examining the middle frontal location. A steeper rise in CBF_*i*_ (*R*^2^ = 0.43; *p* < 0.0001) and CMRO_2*i*_ (*R*^2^ = 0.28, *p* = 0.02) was significantly associated with higher GA at birth, but there was no association between temporal change in OEF and GA (*R*^2^ = 0.03, *p* = 0.74). Similar to our main analyses, there was no significant association with neurological exam at TEA, whether considering the temporal change in CMRO_2*i*_ (OR 1.11; 95% CI 0.95–1.30), CBF_*i*_ (OR 1.07; 95% CI 0.91–1.24), or OEF (OR 1.04; 95% CI 0.96–1.12).

## Discussion

In this cohort of 133 infants born between 29 and 36 6/7 weeks GA, there was a significant association between steeper rises in CMRO_2*i*_ and CBF_*i*_ over time and increasing GA at birth. There was no association between FDNIRS-DCS parameters at TEA or temporal changes and the neurological examination at TEA. To our knowledge, this is the first study assessing the relationship between serial FDNIRS-DCS measurements of brain hemodynamics and oxygen metabolism and a standardized neurological examination at TEA in infants born between 29 and 36 6/7 weeks GA. These findings suggest that FDNIRS-DCS and the neurological exam may be complementary, informing on brain hemodynamics and metabolism and neurological status at TEA, respectively. These data highlight the need to further explore how FDNIRS-DCS could help identifying infants at high neurodevelopmental risk who would benefit from targeted intervention.

There is a growing interest in the use of cerebral NIRS monitoring to improve neurodevelopmental outcomes of infants born preterm. Studies have so far investigated how measures of SO_2_ and OEF obtained in the first days of life are associated with later neurodevelopmental outcomes in infants born very preterm with inconsistent results. Two cohort studies of infants born before 32 weeks GA indicated that a lower cerebral SO_2_ in the first 72 h of life was associated with lower cognitive scores at 24 months CA (Alderliesten et al., 2019; Katheria et al., 2021). In a study of 67 infants born before 32 weeks GA, lower SO_2_ in the first 2 weeks of life was associated with less optimal neurodevelopment at 2–3 years of age, while higher OEF on the first day of life was associated with lower motor scores (Verhagen et al., 2015). Also, a randomized control trial of 115 infants born very preterm assessed the impact of cerebral SO_2_ monitoring on neurodevelopment and found no association between SO_2_ in the first 72 h of life and neurological outcomes at 2 years CA (Plomgaard et al., 2019).

Evidence from diverse clinical settings indicates that FDNIRS-DCS monitoring may provide a more complete assessment of the brain’s state than SO_2_ or OEF alone. This is because conventional NIRS measure of SO_2_ values cannot distinguish whether changes occur in cerebral metabolism or delivery, or both. OEF also reflects a combination of oxygen delivery and consumption, which may limit its interpretation, while FDNIRS-DCS allows to differentiate oxygen demand (metabolism) and supply (flow), and may therefore determine their independent contribution (Lin et al., 2016). In the preterm population, research suggests that CBF_*i*_ and CMRO_2*i*_ may inform more accurately on early cerebral physiological and metabolic changes occurring through brain development (Roche-Labarbe et al., 2012; Lin et al., 2016). A study of 56 infants born between 24 and 37 weeks GA reported that repeated measures of CBF_*i*_ and CMRO_2*i*_ had a better correlation with post-menstrual age compared to SO_2_ (Roche-Labarbe et al., 2012). In a study monitoring infants born extremely preterm until TEA, those with germinal matrix or intraventricular hemorrhage had consistently lower CBF_*i*_ and CMRO_2*i*_, compared to infants with no hemorrhage, but there was no difference in cerebral SO_2_ (Lin et al., 2016). FDNIRS-DCS parameters may also be insightful when neonates undergo clinical intervention that may alter cerebral hemodynamics and metabolism (Levy et al., 2021). A previous study described brain monitoring in infants born extremely preterm before and after patent ductus arteriosus treatment and demonstrated that even if CBF drops significantly, CMRO_2_ can be preserved with a compensatory increase of OEF (Arora et al., 2013). In newborns with single ventricle congenital heart disease, it was shown that CBF_*i*_ and CMRO_2*i*_ monitoring can capture unstable postoperative states and indicate lower values compared with stable states, while SO_2_ may remain the same regardless of the clinical state (Dehaes et al., 2015). Similarly, in a study of newborns with hypoxic ischemic encephalopathy undergoing therapeutic hypothermia, CBF_*i*_ and CMRO_2*i*_ were lower during treatment compared to after, but SO_2_ values were not significantly different between these thermic states (Dehaes et al., 2014). These findings point to a more complete and accurate assessment of cerebral hemodynamics and oxygen metabolism using CBF_*i*_ and CMRO_2*i*_ in comparison to SO_2_ alone.

In the present study, we found that increases in CBF_*i*_ and CMRO_2*i*_ values over time were significantly higher with increasing GA at birth, which is biologically plausible. A previous study using arterial spin labeling perfusion magnetic resonance imaging to quantify CBF found a global and regional increase in CBF values, especially in the frontal area, from 32 to 45 weeks of post-menstrual age, suggesting the high metabolic demand associated with brain growth in the third trimester (Ouyang et al., 2017). Another MRI-based study reported significant increases in global CMRO_2_ between 35 and 42 weeks of post-menstrual age (Liu et al., 2014). Similarly, our study using FDNIRS-DCS indicated that cerebral blood flow and oxygen metabolism increased before TEA, but also demonstrated that this increase occurred at a significantly higher rate with increasing GA at birth. However, first measurements did not correlate with GA at birth. Our findings suggest that the FDNIRS-DCS temporal changes may be more informative than a single timepoint measurement. With the growth and physiological maturation of the newborn brain over time, we would expect an increase in both cerebral blood flow and oxygen metabolism (Roche-Labarbe et al., 2010). With increasing GA, we would also expect that infants would be born with an already more mature and more voluminous brain (Fenton and Kim, 2013), resulting in higher cerebral blood flow and oxygen metabolism. In contrast to CBF_*i*_ and CMRO_2*i*_, we found no association between GA and OEF temporal change. Since OEF represents the fraction of oxygen extracted from arterial blood in the brain, variations in OEF would reflect transient compensatory mechanisms to maintain cerebral oxygenation rather than brain maturation (Brown et al., 2003).

Several factors may explain why FDNIRS-DCS parameters were not associated with the neurological examination at TEA. It is possible that the standardized neurological assessment captured subtle abnormalities that were not reflected on brain hemodynamics and oxygen metabolism, or vice-versa. Also, the neurological examination and FDNIRS-DCS measures may not assess the same processes of infant brain development. FDNIRS-DCS monitoring provides information on brain hemodynamics and oxygen metabolism, which likely reflects brain growth in preterm infants (Roche-Labarbe et al., 2010). A comparative study of brain volumes extracted from structural MRI would help correlating the two modalities for brain growth. In addition, FDNIRS-DCS reflects maturational processes fueled by nutrients such as oxygen, including neuronal proliferation and migration, myelination, and synaptogenesis (Roche-Labarbe et al., 2012; Lin et al., 2013b,^2016^). The assessment of cerebral oxygen metabolism and blood flow evolution before TEA, a critical time window for brain developmental processes (Volpe, 2009), is thus informative. Standardized neurological examinations at TEA mirror the global and structural integrity of the brain and have been shown to correlate with neuroanatomical abnormalities or injuries identified on cerebral MRI in very low birth weight infants (Woodward et al., 2004). The ATGNA provides information on cortical and upper structure integrity such as axial tone, cranial signs, and alertness level, but also considers brainstem function *via* the assessment of primitive reflexes and passive limb flexor tone (Simard et al., 2011). A study including 38 infants born before 32 weeks GA found that lower brainstem, corticospinal and corticopontine pathways volumes on MRI were associated with less optimal ATGNA scores at TEA (Raguž et al., 2021). It was also shown that the ATGNA at TEA identified children at highest risk of poor developmental outcomes at 2 years CA (Simard et al., 2011). Therefore, combining the two modalities, the neurological examination and FDNIRS-DCS, could offer a thorough understanding of the neurological state, and may enhance risk stratification for neurodevelopmental impairment. This hypothesis requires long-term neurodevelopmental follow-up assessment to be confirmed.

Limitations must be acknowledged when interpreting these findings. FDNIRS-DCS data quality may be affected when measurements are performed in infants with small heads, which represents a challenge in preterm populations. The interpretation of our findings is limited to the frontal cerebral physiology as none of the measurements were performed in other cerebral regions, and therefore is not reflecting whole brain physiology. We focused on the middle frontal region in our main analyses to optimize data quality, but sensitivity analyses indicated that associations were similar whether considering the middle or the left and right frontal locations. Also, previous studies demonstrated that results were consistent when considering a single location vs multiple positions (Roche-Labarbe et al., 2012), and showed little variation in FDNIRS-DCS parameters between locations, including no significant difference in CMRO_2*i*_ between the left and right frontal hemispheres (Lin et al., 2013b). The estimation of SO_2_ was based on assumed arterial:venous ratio previously used in premature infants with similar post-menstrual age as well as in newborns and children with congenital heart disease aged <8 years with varying arterial oxygen saturations (Watzman et al., 2000). While this ratio has been used for a broad range of ages, it may vary between birth and TEA, which could introduce a bias when interpreting OEF and CMRO_2*i*_ results. However, ranges of cerebral SO_2_ is narrower from preterm to term gestational ages, compared with the more variable mesenteric or renal SO_2_ values (Alderliesten et al., 2016; Van Meurs, 2016), thus limiting the magnitude of bias. The clinical state and activity level of the newborn at time of measurement may have affected the results. We performed repeated FDNIRS-DCS measurements and considered temporal changes in our analyses, but timepoint measurements may be influenced by transient variations that do not fully reflect the baseline cerebral state. Although the ATGNA provides results that are stable over time (Simard et al., 2010), a one-time neurological examination at TEA may capture transient neurological signs or miss abnormalities that would only manifest later. Future studies examining the association between FDNIRS-DCS parameters and serial neurological examinations are needed.

## Conclusion

This study shows that temporal changes in CMRO_2*i*_ and CBF_*i*_ are associated with GA at birth and may represent potential indicators of brain development and growth in preterm infants. At TEA, there was no association between FDNIRS-DCS parameters and the neurological examination. Whether these two measures provide different, perhaps complementary information on the infant brain needs to be further investigated.

## Data availability statement

The raw data supporting the conclusions of this article will be made available by the authors, without undue reservation.

## Ethics statement

The studies involving human participants were reviewed and approved by the Institutional Review Board of the Comité d’Éthique de la Recherche at CHU Sainte-Justine, named by the Quebec Government (#FWA00021692), in accordance with Quebec and Canada Laws, and the Code of Federal Regulations in the United States. Written informed consent to participate in this study was provided by the participants’ legal guardian/next of kin.

## Author contributions

GC-C, M-NS, MD, and TML conceived and designed the study. OB, RC, and MD contributed to the FDNIRS-DCS assessment. M-MG, MG, and TML performed the neurological examinations. CB contributed to the clinical data collection. GC-C and OL performed the statistical analyses with input from M-NS, MD, and TML. GC-C drafted the original manuscript with critical revisions from M-NS, AMN, MD, and TML. All authors contributed to the acquisition, analysis, and interpretation of data, read, and approved the final manuscript.
