# Supplementary material for: Associations between neurological examination at term-equivalent age and cerebral hemodynamics and oxygen metabolism in infants born preterm
Source: Front Neurosci. 2023 Mar 2;17:1105638. doi: 10.3389/fnins.2023.1105638 (PMC10017489; doi:10.3389/fnins.2023.1105638)
Supplement: Supplementary file 1 [file Data_Sheet_1.docx]

**Supplementary Figure 1**. Associations between first measure of (A) cerebral blood flow index (CBF_i_), (B) cerebral metabolic rate of oxygen consumption index (CMRO_2i_), and (C) cerebral oxygen extraction fraction (OEF) with gestational age at birth (GA). For each parameter, a linear fit (blue line) and corresponding correlation coefficient (R^2^) and *p*-value are provided.

**Supplementary Table 1**. Neonatal characteristics of participants who completed or not the study.

|  | Lost to follow-up (n = 17) | Included in the study (n = 133) |
| --- | --- | --- |
| Gestational age, mean (SD), weeks | 33.6 (2.0) | 33.3 (1.9) |
| Birth weight, mean (SD), g | 2117.4 (537.9) | 1831.0 (549.4) |
| Birth weight Z-Score, mean (SD) | -0.0 (0.6) | -0.6 (1.1) |
| Head circumference, mean (SD), cm | 30.3 (2.3) | 29.6 (2.1) |
| Male, n (%) | 8 (47.1) | 73 (54.9) |
| Multiple birth, n (%) | 2 (11.8) | 41 (30.8) |
| Antenatal corticosteroids, n (%) | 11 (64.7) | 107 (80.5) |
| Urgent cesarean section, n (%) | 5 (29.4) | 65 (48.9) |
| Surfactant administration, n (%) | 2 (11.8) | 24 (18.0) |

SD: Standard deviation.

**Supplementary Table 2**. Associations between cerebral SO_2_ measures and temporal changes and neurological examination at term-equivalent age.

| Odds ratios (95% confidence interval) | | | |
| --- | --- | --- | --- |
|  | First SO_2_ measure^a^ | SO_2_ measure at TEA | SO_2_ temporal change |
| All participants | 1.02 (0.94-1.11) | 1.02 (0.95-1.10) | 0.90 (0.77-1.05) |
| Stratified by gestational age |  |  |  |
| 29-32 weeks | 1.06 (0.90-1.25) | 1.05 (0.90-1.23) | 0.58 (0.22-1.51) |
| 33-36 weeks | 1.01 (0.91-1.11) | 1.00 (0.91-1.10) | 0.91 (0.78-1.07) |
| Stratified by sex |  |  |  |
| Females | 0.97 (0.87-1.08) | 1.06 (0.94-1.19) | 1.01 (0.81-1.27) |
| Males | 1.08 (0.95-1.23) | 1.00 (0.90-1.10) | 0.81 (0.62-1.05) |

^a^Odds ratios with first SO_2_ measures are adjusted for post-menstrual age at time of measurement.

SO_2_: Oxygen hemoglobin saturation; TEA: Term-equivalent age.
