# Supplementary figures and images for: Associations between neurological examination at term-equivalent age and cerebral hemodynamics and oxygen metabolism in infants born preterm
Source: Front Neurosci. 2023 Mar 2;17:1105638. doi: 10.3389/fnins.2023.1105638 (PMC10017489; doi:10.3389/fnins.2023.1105638)

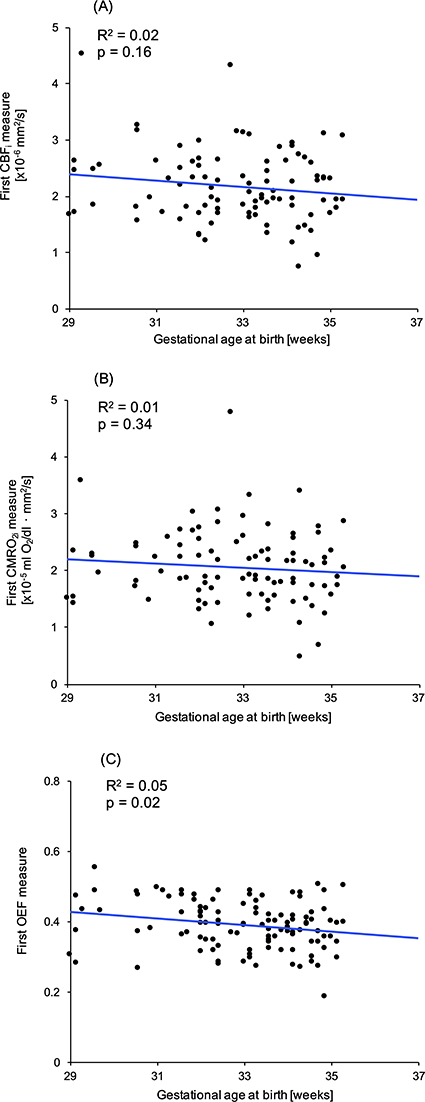

Supplement: Supplementary file 2 [file Image_1.TIFF]
